# Therapeutic Hypothermia Combined with Hydrogen Sulfide Treatment Attenuated Early Blood–Brain Barrier Disruption and Brain Edema Induced by Cardiac Arrest and Resuscitation in Rat Model

**DOI:** 10.1007/s11064-022-03824-5

**Published:** 2022-11-24

**Authors:** Shenquan Cai, Qian Li, Jingjing Fan, Hao Zhong, Liangbin Cao, Manlin Duan

**Affiliations:** 1 Department of Anesthesiology, Affiliated Jinling Hospital, Medical School of Nanjing University, No.305 East Zhongshan Road, Nanjing, 210002 Jiangsu China; 2 Department of Anesthesiology, Jiangning Hospital Affiliated to Nanjing Medical University, Nanjing, Jiangsu China

**Keywords:** Cardiac arrest, Cardiopulmonary resuscitation, Hydrogen sulfide, Therapeutic hypothermia, Blood–brain barrier

## Abstract

Brain injury remains a major problem in patients suffering cardiac arrest (CA). Disruption of the blood–brain barrier (BBB) is an important factor leading to brain injury. Therapeutic hypothermia is widely accepted to limit neurological impairment. However, the efficacy is incomplete. Hydrogen sulfide (H_2_S), a signaling gas molecule, has protective effects after cerebral ischemia reperfusion injury. This study showed that combination of hypothermia and H_2_S after resuscitation was more beneficial for attenuated BBB disruption and brain edema than that of hypothermia or H_2_S treatment alone. CA was induced by ventricular fibrillation for 4 min. Hypothermia was performed by applying alcohol and ice bags to the body surface under anesthesia. We used sodium hydrosulphide (NaHS) as the H_2_S donor. We found that global brain ischemia induced by CA and cardiopulmonary resuscitation (CPR) resulted in brain edema and BBB disruption; Hypothermia or H_2_S treatment diminished brain edema, decreased the permeability and preserved the structure of BBB during the early period of CA and resuscitation, and more importantly, improved the neurologic function, increased the 7-day survival rate after resuscitation; the combination of hypothermia and H_2_S treatment was more beneficial than that of hypothermia or H_2_S treatment alone. The beneficial effects were associated with the inhibition of matrix metalloproteinase-9 expression, attenuated the degradation of the tight junction protein occludin, and subsequently protected the structure of BBB. These findings suggest that combined use of therapeutic hypothermia and hydrogen sulfide treatment during resuscitation of CA patients could be a potential strategy to improve clinical outcomes and survival rate.

**Supplementary Information:**

The online version contains supplementary material available at 10.1007/s11064-022-03824-5.

## Introduction

Brain injury remains a major problem in patients suffering CA [1]. Despite of the advances in CPR methods, the survival rate remains low, and many survivors experience long-term neurological dysfunction [2]. The BBB is formed by endothelial cells of the cerebral vasculature and prevents extravasation of blood products into the brain to protect neural tissue and maintain a homeostatic environment [3]. BBB breakdown has been documented in animal models and patients with CA [4, 5], BBB breakdown is involved in the initiation of transcriptional changes in the neurovascular unit that ultimately lead to delayed neuronal dysfunction and cell death; the neurovascular unit, which is composed of neurons, astrocytes, in addition to the specialized endothelial cells, mural cells, and the basement membrane [3, 6]. Tight junctions (TJs) constitute the junction complex of the BBB, the TJs are present at the sites of fusion involving the outer surface of the plasma membrane of adjacent endothelial cells, and the TJs form a metabolic and physical barrier to restrict the paracellular permeability [3]. Disruption of the structure of BBB leads to extravasation of macromolecular proteins, and small molecule solutes into extracellular space, resulting in vasogenic brain edema and cell death [7]. Matrix metalloproteinase-9 (MMP-9) is reported to degrade the TJs complex, leading to BBB leakage, vasogenic brain edema and brain damage [8].

Therapeutic hypothermia is widely accepted as an effective method to improve survival and limit neurological outcomes in patients who achieve return of spontaneous circulation (ROSC) after CA [9–11]. Models of CA have shown that hypothermia protects the BBB and prevents edema formation [12]. Despite that, hypothermia alone is difficult to achieve the expected recovery effect in clinical practice [13, 14]. Thus, the development of alternative approaches with or without hypothermia is an unmet medical need in ameliorating the prognosis of post-CA patients. H_2_S has been recognized as the third gaseous signaling molecule, with a relatively small molecular mass, which allows it to traverse the cell membrane freely [15]. H_2_S has been referred as a neuromodulator and neuroprotectant in the central nervous system, produces anti-oxidant, anti-inflammatory, and anti-apoptotic effects in cerebral injury [16, 17]. In the field of CA and CPR, research of H_2_S in cerebral injury after CA has gradually increased [18–20]. Geng et al. demonstrated H_2_S improved the integrity of BBB, mitigated brain edema; improved neurological outcome and 14-days survival rate in rats after CA and resuscitation [18]. These findings suggest that H_2_S could protect the BBB integrity after resuscitation. Therefore, this study will test the hypothesis whether the combination of hypothermia and H_2_S after resuscitation was more beneficial than that of hypothermia or H_2_S treatment alone, and examine the possible mechanisms for the effects.

## Materials and Methods

### Animal Surgical Procedures

Male Sprague–Dawley rats, weighing 280 to 320 g, 7–8 weeks old, were provided by the Animal Center of Jinling Hospital, Nanjing, China. All rats were housed in controlled room on a 12-h light–dark cycle and fed a standard laboratory diet. This study was approved by the Ethics Committee of Jinling Hospital and was performed in accordance with the guidelines for the use of experimental animals by the National Institutes of Health. For experiments, animals were fasted overnight except for free access to water. Animals were anesthetized with intraperitoneal injection of 2% sodium pentobarbital (50 mg/kg) and intubated tracheally with a 14-gauge cannula. Polyethylene catheters were inserted into the left femoral artery and vein and flushed intermittently with saline solution which containing 2.5 IU/ml bovine heparin. The arterial catheter line was connected to a pressure transducer (PT-100, Chengdu Taimeng Software Co.LTD, China) to measure mean aortic pressure (MAP) and the venous catheter was used for medical administration, and the electrocardiogram was recorded by subcutaneous needle electrodes. Core temperature was monitored by a rectal temperature probe (BAT-10, Physitemp Instruments Inc) throughout the experiment to ensure appropriate temperature management.

### Cardiac Arrest and Cardiopulmonary Resuscitation

CA and CPR in rats were performed as previously described with some slight modifications [21, 22]. CA was induced using a 5F pacing catheter, inserted orally into the esophagus of the rats approximately 7 cm in depth. Continuous cardiac pacing was conducted and maintained for 1 min (frequency: 25 Hz; intensity: 25 V; stimulus duration width: 10 ms) to induce CA. A stimulation pause was then initiated for a few seconds (1–3 s) to observe the change of ECG, as soon as the rhythm reverted spontaneously, an additional 30-s stimulation was performed immediately until the ventricular fibrillation reappeared and persisted. Cardiac arrest was confirmed by the absence of autonomous respiration and MAP < 20 mmHg. After 4 min of CA, CPR was started, including chest compressions and ventilation conducted by animal ventilator (Beijing zhong shi di chuang science and technology development Co.,LTD, China) (Respiratory parameters: oxygen concentration: 100%, ventilation frequency: 60 breaths/min, tidal volume: 6 ml/kg). A dose of epinephrine (100 μg/kg) and 5% (w/v) sodium bicarbonate (1.0 ml) were injected via the femoral vein simultaneously. After 1 min of CPR, the animals were counter shocked with a 7-J DC current delivered to the heart through the transoesophageal cardiac pacing electrode. Additional doses of sodium bicarbonate were administered according to arterial blood gas analysis performed at 10 and 30 min after ROSC (the additional required dose was calculated by the formula: bicarbonate (mmol) = (− 2.3—the actual measured value of base excess) * 0.25 * body weight (kg). ROSC was defined as an organized cardiac rhythm with a MAP > 60 mmHg, which was sustained continuously for at least 5 min. If the spontaneous circulation of the rats was not restored after 10 min with the above treatment, CPR was considered a failure.

After ROSC, rats were mechanically ventilated and invasively monitored for 6 h in maintaining the target temperature. Blood samples were drawn for blood gases, glucose, and lactate measurements at baseline and 10 and 30 min after ROSC. Rats were then weaned from the ventilator, tracheally extubated, and returned to their cages with easily accessible food and water. The survival time after CPR was recorded up to 7 days.

### Experimental Protocol

**Fig. 1:**
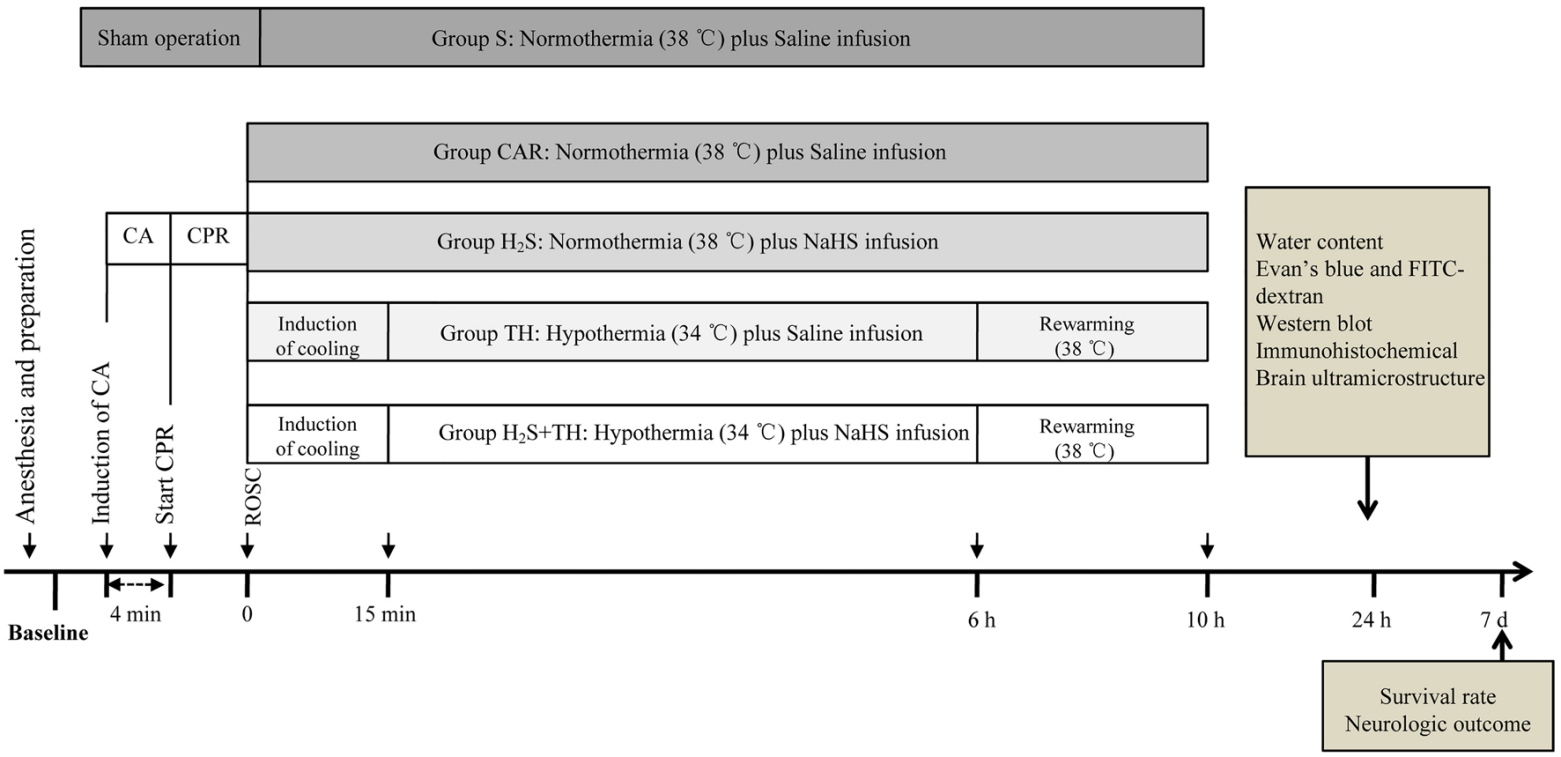
Experimental time line. Rats were subjected to CA induced by electrical stimulation or sham operation. After four minutes of CA, rats were resuscitated by chest compression and mechanical ventilation. After successful ROSC, rats were randomized to one of four groups: Group S, Group CAR, Group H_2_S, Group TH and Group H_2_S + TH

The experimental time line is presented in Fig. 1. After successful resuscitation, the animals were randomized to one of the four groups: cardiac arrest and resuscitation group (CAR), sodium hydrosulfide group (H_2_S), therapeutic hypothermia group (TH), sodium hydrosulfide combined with therapeutic hypothermia group (H_2_S + TH). NaHS (Sigma-Aldrich, St. Louis, MO, USA) was freshly diluted in normal saline to the desired concentration (0.3 mg/ml) before administration. The NaHS was infused intravenously with an initial loading dose of 0.5 mg/kg at the start of CPR, followed by a maintenance infusion of NaHS (1.5 mg·kg^−1^·h^−1^) until 6 h after ROSC. This dosage was based on a previous study with minor modification [23]. Hypothermia was performed as follows: we initiated cooling after ROSC by applying alcohol and ice bags to the body surface under anesthesia. Rectal temperature was reduced to 34 °C within 15 min of initiating reperfusion, maintained for 6 h by exposing the rat to ice bag or a heat lamp, and the distance between the rats and the lamp was adjusted to maintain target temperature. Hypothermic rats were rewarmed beginning at 6 h after ROSC at a rate of approximately 1 °C /h over 4 h with a heat lamp until rectal temperature reached 38.0 °C. Sham treated animals underwent all procedures except CA and resuscitation, received an equivalent volume of normal saline, and the rectal temperature were maintained at 38.0 °C. Throughout the experiment, a total number of 222 rats were used, 5 rats died during the operation, and 17 rats died due to the failure of ROSC, and 10 rats died before the test. Therefore, 190 rats were involved in the statistics. The whole experiment consists of two parts. In part one, arterial blood for blood gas analysis was obtained at baseline at 10 and 30 min after ROSC in each group, the neurological function was evaluated at 1 day, 3 day and 7 day after ROSC and their survival rate was monitored up to 7 days after ROSC (n = 5 for the sham group; n = 15 for each group of the other 4 groups). In part two, the brain edema, BBB integrity, the protein expression and the BBB ultrastucture alteration were measured at 24 h after ROSC (n = 25 for each group).

### Assessment of Survival Rate and Neurologic Outcome

The survival rate was monitored up to 7 days after ROSC or sham operation, and the neurological function was evaluated at 1, 3, and 7 day after ROSC or sham operation. A modified tape removal test described previously was conducted to evaluate neurologic outcome [24]. In brief, 10 mm by 12 mm adhesive tapes were affixed to each of the animal’s front paws. The time to remove both adhesive tapes was recorded. The test was truncated at 180 s and all times > 179 s were recorded as ‘180 s’. Before the experiment, all animals were familiarized with the neurologic test for 3 consecutive days. All evaluations were performed by the same investigator who was masked to treatment.

### Determination of Brain Water Content

Cortical and hippocampal water content was determined by wet-dry method at 24 h after ROSC or sham operation. The left hemisphere of the brain was used for here, right cerebral hemisphere was for western blot analysis. Brain tissue was immediately divided into cortex and hippocampus after decapitation and weighed to obtain the wet weight. The tissues were subjected to slow evaporation in a laboratory oven (80 °C) for 72 h and reweighed to determine the dry weight. Brain water content (%) was calculated as (wet weight–dry weight)/(wet weight) × 100%.

### Evaluation of Blood–Brain Barrier Permeability

Evans blue (EB) and fluorescein isothiocyanate–dextran (FITC-dextran) used to assess macromolecular proteins and small solute permeability at 24 h after ROSC or sham operation respectively. EB dye (Sigma Chemical Co., St. Louis, USA; 2% solution in saline, 2 ml/kg), which can bind to albumin (molecular weight, about 68 kDa) and FITC–dextran (Sigma Chemical Co., St. Louis, USA; 5% solution in saline, 2 ml/kg) (average molecular weight, 4 kDa) were administered intravenously and allowed to circulate for 30 or 2 min, respectively. Rats were then administered transcardial saline to remove intravascular EB or FITC-dextran. The brains were removed and rinsed with phosphate—buffered saline, and two 4-mm wide coronal slices were made. The cerebral cortex above the rhinal fissure from the first slice and the hippocampus from the second slice were dissected as shown according to previous studies [25]. After weighing, the cortex and hippocampus were homogenized in 50% trichloroacetic acid. For EB measurement, samples were centrifuged at 21,000 g for 20 min. The supernatant was collected, and EB per weight of sample was measured at 620 nm with a spectrophotometer, and quantified by a series of standard EB solution (100–1000 ng/mL). For FITC-dextran measurement, samples were centrifuged at 10,000 g for 20 min, the supernatant was collected, and FITC–dextran fluorescence (ng/mL) was measured at 538 nm using 485 nm excitation (PerSeptive Biosystems, USA). Total fluorescence of each sample was calculated from concentrations of external standards (100–8000 ng/mL) and presented as percentage of change from the sham group.

### Ultrastructure Alteration of BBB

BBB ultrastructure alterations were observed by a transmission electron microscopy. The rats were decapitated at 24 h after reperfusion, the hippocampi were cut into 1 mm^3^, fixed in 1% freshly made paraformaldehyde and 2% glutaraldehyde for 24 h at 4 °C, washed with 0.1 mol/L phosphate buffer for 3 times. Then the samples were post-fixed in 1% osmium tetroxide in 0.1 mol/L phosphate buffer for 2 h at 4 °C. After fixation, the samples were dehydrated in grade acetone and embedded in Epon 812. The ultra-thin sections of hippocampus were stained with uranium acetate and lead citrate and then examined with a transmission electron microscope (JEM 1230, JEOL, Japan). The luminal membrane segments of the capillary here selected in which the membrane was clearly visible. Then the optical density was measured along a line perpendicular to the membrane, starting at the capillary lumen and continuing through the whole TJs and the membrane. The thickness of the TJs was then defined as the distance between the point at the luminal side at which 50% of the maximal optical density of the TJs was measured and the translucent center of the cytoplasmic membrane.

### Western Blot Analysis

Western blot analysis was used to assess expression of TJs occludin in the cortex and hippocampus, the samples were harvested at 24 h after ROSC, frozen in liquid nitrogen and stored at − 80 °C for western blot analysis. Protein homogenates of samples were prepared by rapid homogenization in Tissue Extraction Reagent II (Invitrogen Corporation, Carlsbad, USA), according to the manufacturer’s instructions. After homogenization, tissue samples were centrifuged at 15 000 g for 20 min at 4 °C. Protein concentration was determined using a BCA protein assay kit (Bio-Rad, Hercules, USA). Proteins (30 μg) were electrophoresed on 12% Tris–glycine gels, and then transferred onto polyvinylidene difluoride membranes. Membranes were incubated with primary anti-occludin (1:1000, Abcam, Cambridge, USA), anti-β-actin (1:2000, Abcam, Cambridge, USA) followed by incubation with horseradish peroxidase-conjugated secondary antibodies (1:5000, Cell Signaling Technology, USA). Protein bands were visualized with an enhanced luminescence reagent (Millipore) and photographed with ChemiDoc XRS + (Bio-Rad, Hercules, USA). Final results were normalized to β-actin and expressed as the ratios of target proteins/β-actin.

### Immunohistochemical Procedures

Immunohistsochemical analyses were performed as previously described [26]. Rats were perfused through the left ventricle of the heart with phosphate—buffered saline and then with 4% paraformaldehyde in 0.01 mol/L phosphate—buffered saline. Hippocampi were fixed in 4% paraformaldehyde and embedded in paraffin wax prior to sectioning. The fixed brains were immersed in 20% sucrose in phosphate—buffered saline overnight, then tissues were sectioned at 4-μm thickness. After antigen retrieval treatment and 5% BSA blocking, sections were incubated overnight at 4 °C with the primary antibody anti-MMP-9 (1:200, Abcam, Cambridge, USA), followed by rabbit anti-rat IgG-HRP antibody (1:100, Cell Signaling Technology, USA) at room temperature for 2 h. Thereafter, the sections were incubated with streptavidin-peroxidase (Fuzhou Maixin Biotech Co. Ltd., China), and visualized with diaminobenzidine stain. For each tissue, four fields (× 400) were selected for each section, and the number of positive cells was counted. The average positive cells of each slice were obtained by dividing the sum of the positive cells counted from each field by four. The average of each sample was used for statistical analysis.

### Statistical Analysis

SPSS 16.0 software (SPSS Inc., Chicago, IL) were used for statistical analyses. Survival was expressed as a percentage and the Kaplan–Meier survival curves were compared using log-rank testing, and we used a Bonferroni correction for multiple comparisons. Date from the tape removal test were presented as the median (quartiles); and analyzed using the Kruskal–Wallis test, a Nemenyi test was performed when the overall P value was significant. Other data were presented as the mean ± SD. Normal distribution data were confirmed using the Kolmogorov–Smirnov test, and analyzed by one-way analysis of variance (ANOVA) followed by Bonferroni test for intergroup comparisons. The Bonferroni-adjusted P value was defined such that the raw P value multiplies the number of comparisons. P < 0.05 was considered statistically significant.

## Results

### Physiological Parameters and Therapies During Cardiopulmonary Resuscitation

At baseline, physiological parameters including arterial blood gas samples, heart rate, blood pressure and core body temperature were in the normal range, there were no differences between the groups. During induction of CA, no significant differences were observed with regard to the duration of CPR time, the number of defibrillations, the dose of adrenaline or the rate of successful resuscitation between the groups. There were no significant differences in arterial pH, PaO_2_, PaCO_2_, base excess, hematocrit, glucose, blood pressure among the groups at 10 and 30 min after resuscitation. (Supplement Table 1, 2).

### Time Course of Rectal Temperature

During CA and CPR, rectal temperature in rats was maintained at 38 °C. In the TH and H_2_S + TH groups, rectal temperature was rapidly decreased to 34 °C within 15 min with surface cooling. Subsequently, temperature was well maintained for 6 h. In the CAR and H_2_S groups, rectal temperature was maintained at 38 °C during the experiment. During the period of rewarming, temperature was increased at approximately 1 °C per hour (Supplement Fig. 1).

### Hypothermia and Hydrogen Sulfide Improved the Survival Rate

**Fig. 2:**
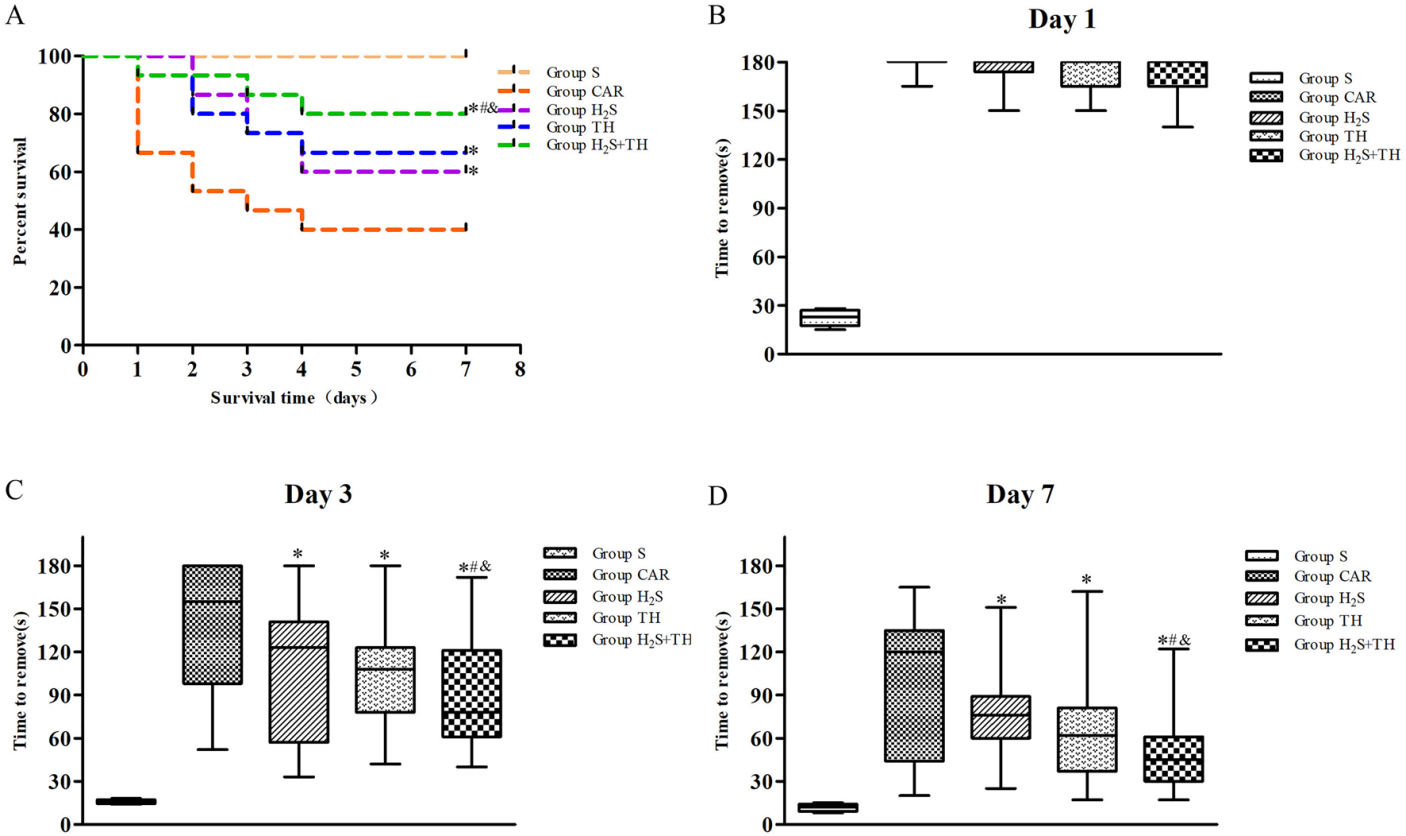
Effects of hypothermia and hydrogen sulfide treatment on survival rates and neurological function of rats with CA and resuscitation. **A** Kaplan–Meier plot of cumulative survival 7 days after CA and resuscitation in Group S (n = 5), Group CAR (n = 15), Group H_2_S (n = 15), Group TH (n = 15), and Group H_2_S + TH (n = 15). Data was presented as a percentage. **B**–**D** Time needed in the tape removal test at day 1, day 3, and day 7 after resuscitation. Date was presented as median (quartiles). ^*^P < 0.05 versus Group CAR, ^#^*P* < 0.05 versus Group H_2_S, ^&^*P* < 0.05 versus Group TH

CA and CPR rats were followed up to 7 days, and survival rate was recorded every day. As shown in Fig. 2, the survival rate 7 days after ROSC was 100% (5/5) in the sham group, 40% (6/15) in the CAR group, 60% (9/15) in the H_2_S group, 67% (10/15) in the TH group, and 80% (12/15) in the H_2_S + TH group. The difference of survival rate in groups was statistically significant (log rank test,* P* < 0.05). Compared with CAR group, we found that immediately after resuscitation, hypothermia treatment or H_2_S applied individually improved the survival rate 7 days after resuscitation (*P* < 0.05). And the use of H_2_S with concurent treatment with hypothermia could further increased the effectiveness of hypothermia alone (*P* < 0.05). The results indicated that the effect of combined of hypothermia and H_2_S treatment on the short-term survival rate after CPR was more beneficial than the individual treatment.

### Hypothermia and Hydrogen Sulfide Improved the Neurological Function

A modified tape removal test described previously was conducted to evaluate neurologic outcome. Date from the tape removal test were presented as the median (quartiles); and analyzed using the Kruskal–Wallis test. The difference of tape removal time in groups was statistically significant (*P* < 0.05). As shown in Fig. 2, we found that all resuscitated animals remained severely impaired at day 1 after ROSC, the time for most animals removed the adhesive tapes were near 180 s, there were no differences in the four groups of rats that experienced CA at day 1 after ROSC. With the prolongation of time, the tape removal time was gradually shortened. Compared with CAR group, the time needed on day 3 was decreased in the H_2_S group, TH group and H_2_S + TH group (*P* < 0.05). Test on day 7 also revealed a significantly better performance of the group treated with hypothermia and H_2_S. Moreover, the time needed in the H_2_S + TH group was shorter than the H_2_S group (*P* < 0.05) and TH group (*P* < 0.05). The results indicated that neurological function was improved by treatment with hypothermia or H_2_S during CPR in post-CA rats. Combined treatment of hypothermia with H_2_S had additive effect on neurological function after ROSC.

### Hypothermia and Hydrogen Sulfide Diminished Brain Edema

**Fig. 3:**
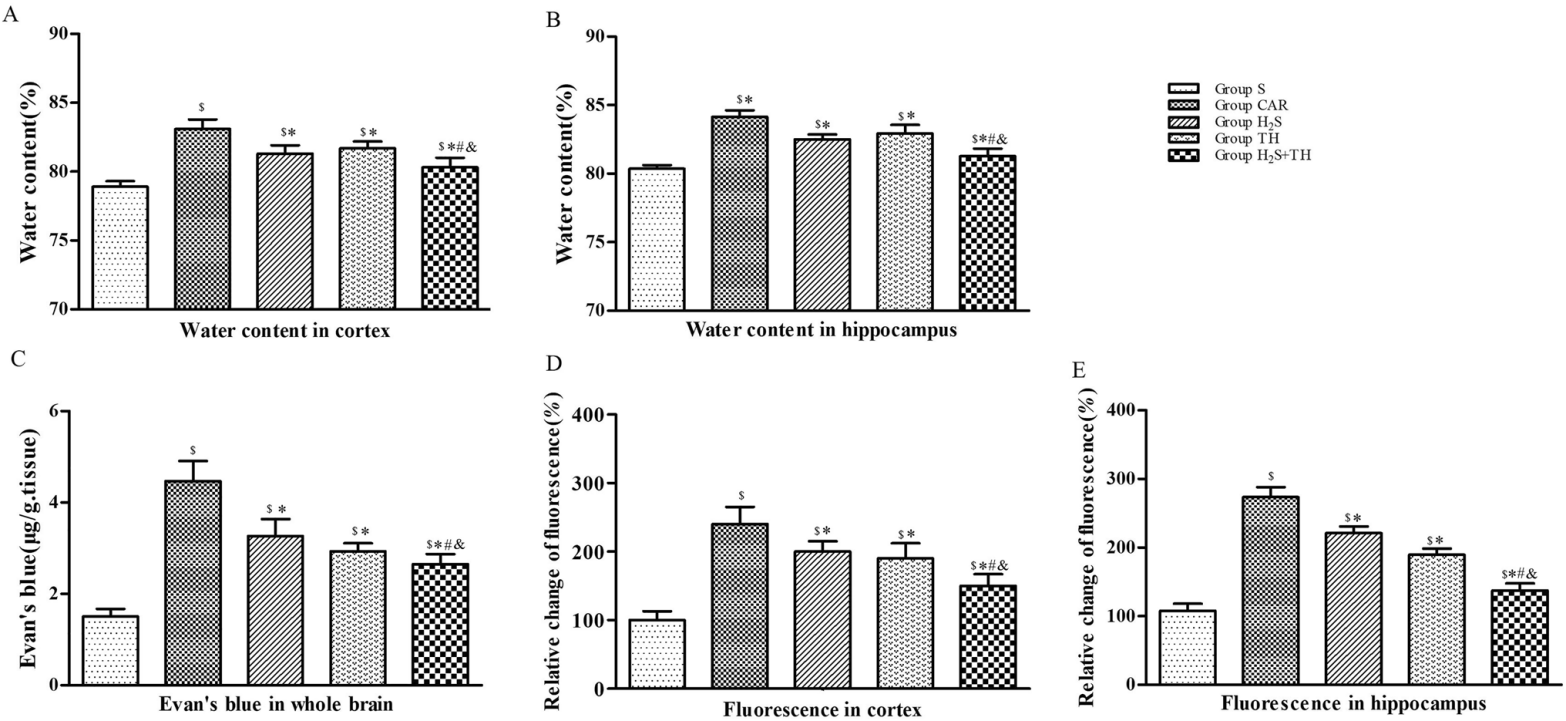
Effects of hypothermia and hydrogen sulfide treatment on brain edema and BBB integrity of rats with CA and resuscitation. **A**, **B** Brain water content in the cortex and hippocampus. Brain water content, an indicator of brain edema, was measured with wet-dry method at 24 h after resuscitation or sham operation (n = 5 rats per group). **C**–**E** EB and FITC–dextran permeability in the brain. BBB permeability was evaluated using EB in the whole brain, and FITC–dextran permeability in the cortex and hippocampus at 24 h after resuscitation or sham operation (n = 5 rats per group). Data are presented as mean ± SD. ^$^*P* < 0.05 versus Group S, ^*^P < 0.05 versus Group CAR, ^#^*P* < 0.05 versus Group H_2_S, ^&^*P* < 0.05 versus Group TH

The water content of cortex and hippocampus was measured at 24 hours after ROSC or sham operation. The difference of water content of cortex and hippocampus in groups was statistically significant (*F*_*(4,20)*_=*158.7; P* < *0.05*) and (*F*_*(4,20)*_=*185.2; P* < *0.05*). Compared with sham group, the water content was significantly increased in the CAR group, H_2_S group, TH group and H_2_S+TH group (*P* < 0.05). Compared with group CAR, the water content of cortex and hippocampus were reduced in the H_2_S group, TH group and H_2_S+TH group (*P* < 0.05), and water content in the H_2_S+TH group was less than the H_2_S group (*P* < 0.05) or TH group (*P* < 0.05). These results indicated that H_2_S treatment combined with hypothermia had additive effect on ameliorating brain edema after CA and CPR (Figure 3).

### Hypothermia and Hydrogen Sulfide Decreased the Blood–Brain Barrier Permeability

Since BBB permeability is important to BBB function, we check whether BBB permeability would be altered after CA and hypothermia or H_2_S treatment. BBB permeability was assessed in the brain using EB and FITC-dextran extravasation assays at 24 h after ROSC or sham operation. The difference of EB extravasation in the whole brain in groups was statistically significant (*F*_*(4,20)*_ = *264.8; P* < *0.05*). Compared with the sham group, EB extravasation in the whole brain was significantly increased in the CAR group, H_2_S group, TH group and H_2_S + TH group (*P* < 0.05). In contrast, compared with CAR group, EB extravasation was reduced in the H_2_S group, TH group and H_2_S + TH group (*P* < 0.05), and EB extravasation in the H_2_S + TH group was less than the H_2_S group (*P* < 0.05) or TH group (*P* < 0.05). We further examined the FITC–dextran permeability of cortex and hippocampus. The difference of FITC–dextran extravasation of cortex and hippocampus in groups was statistically significant (*F*_*(4,20)*_ = *228.7; P* < *0.05*) and (*F*_*(4,20)*_ = *200.4; P* < *0.05*). FITC–dextran extravasation in the cortex and hippocampus at 24 h after ROSC in the CA groups were significantly greater than in the sham group (*P* < 0.05). Compared with CAR group, FITC–dextran extravasation was reduced in the H_2_S group, TH group and H_2_S + TH group (*P* < 0.05), and FITC–dextran extravasation in the H_2_S + TH group was less than the H_2_S group (*P* < 0.05) or TH group (*P* < 0.05). (Fig. 3).

### Hypothermia and Hydrogen Sulfide Reduced the Degradation of Occludin

**Fig. 4:**
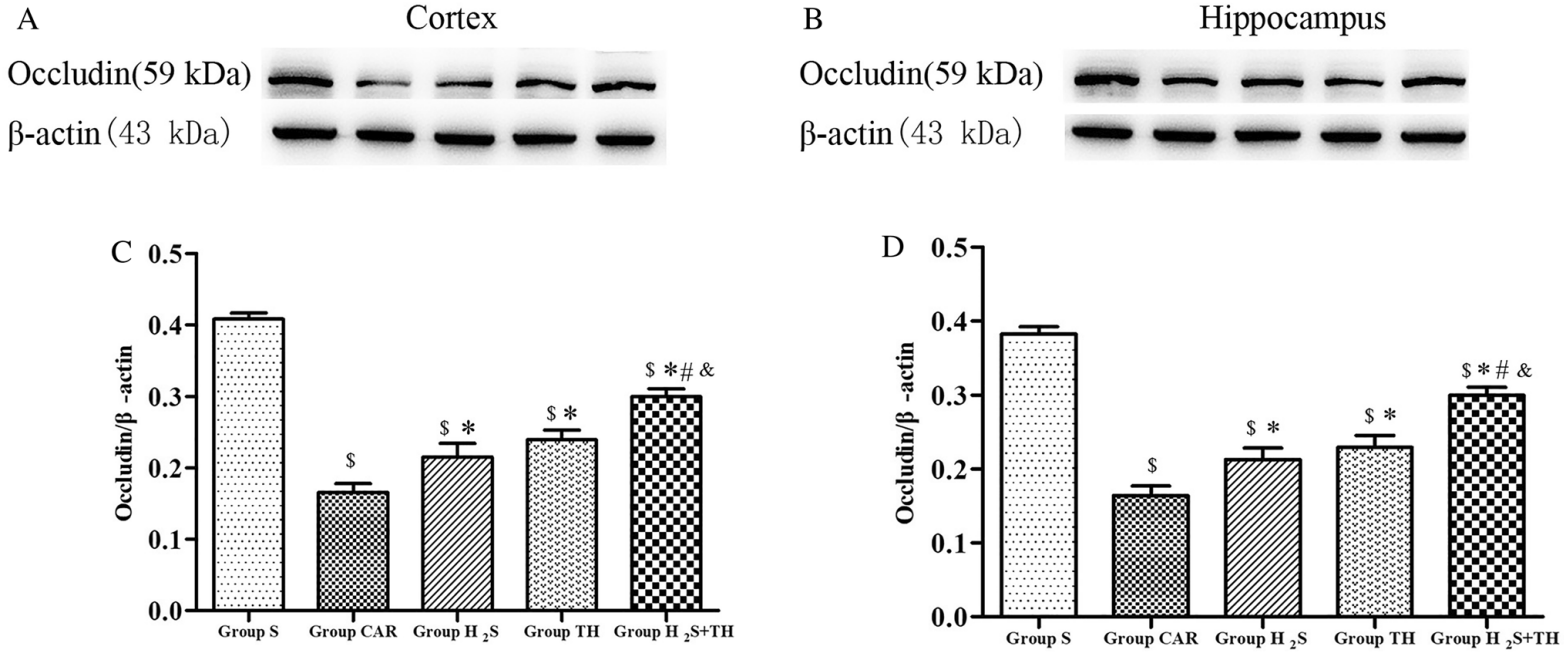
The expression of occludin protein in the cortex and hippocampus at 24 h after resuscitation among groups (n = 5 rats per group). **A**, **B** Representative western blot images of occludin. **C**–**D** The bars of semi-quantitative. Results are expressed as the ratio of occludin and β-actin among groups. Data are presented as mean ± SD. ^$^*P* < 0.05 versus Group S, ^*^P < 0.05 versus Group CAR, ^#^*P* < 0.05 versus Group H_2_S, ^&^*P* < 0.05 versus Group TH

To elucidate the possible mechanisms underlying the BBB alterations that occur after CA, we studied expression of TJs protein occludin, in the cortex and hippocampus obtained at 24 h after ROSC. The difference of the expression of TJs protein occludin in groups was statistically significant in the cortex (*F*_*(4,20)*_ = *125.3; P* < *0.05*) and hippocampus (*F*_*(4,20)*_ = *144.6; P* < *0.05*). As shown in Fig. 4, compared to sham group, the level of occludin in the cortex was significantly decreased in the CAR group, H_2_S group, TH group and H_2_S + TH group (*P* < 0.05). In contrast, compared with CAR group, the level of occludin was increased in the H_2_S group, TH group and H_2_S + TH group (*P* < 0.05), and the level of occludin in the H_2_S + TH group was more than the H_2_S group (*P* < 0.05) or TH group (*P* < 0.05).The level of occludin changes in the hippocampus were similar to those in the cortex.

### Hypothermia and Hydrogen Sulfide Suppressed the Matrix Metalloproteinase-9 Expression

**Fig. 5:**
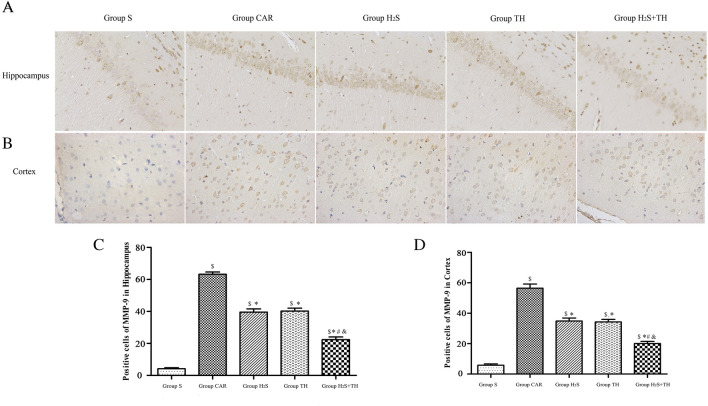
MMP-9 immunohistochemical staining and positive cell counts in the cortex and hippocampus in rats at 24 h after resuscitation (n = 5 rats per group). **A**, **B** MMP-9 immunohistochemical staining in the cortex and hippocampus, respectively. **C**, **D** MMP-9 positive cell counts in the prefrontal cortex and the cornu ammonis (CA-1) area of the hippocampus. Data are presented as mean ± SD. ^$^*P* < 0.05 versus Group S, ^*^P < 0.05 versus Group CAR, ^#^*P* < 0.05 versus Group H_2_S, ^&^*P* < 0.05 versus Group TH

To elucidate the possible molecular mechanisms underlying the BBB disruption that occur after resuscitation, we researched the MMP-9 expression in the brain cortex and hippocampus. The difference of the expression of MMP-9 in groups was statistically significant in the cortex (*F*_*(4,20)*_ = *355.3; P* < *0.05*) and hippocampus (*F*_*(4,20)*_ = *391.6; P* < *0.05*). As shown in Fig. 5, compared to sham group, the expression of MMP-9 in the cortex was significantly up-regulated in CAR, H_2_S, TH and H_2_S + TH groups (*P* < 0.05). In contrast, compared with CAR group, the expression of MMP-9 was decreased in the H_2_S group, TH group and H_2_S + TH group (*P* < 0.05), and expression of MMP-9 in the H_2_S + TH group was decreased more than the H_2_S group (*P* < 0.05) or TH group (*P* < 0.05). Changes of the MMP-9 expression trend in the hippocampus were similar to those in the cortex.

### Effect of Hypothermia and Hydrogen Sulfide on Ultrastructure Alteration of BBB

**Fig. 6:**
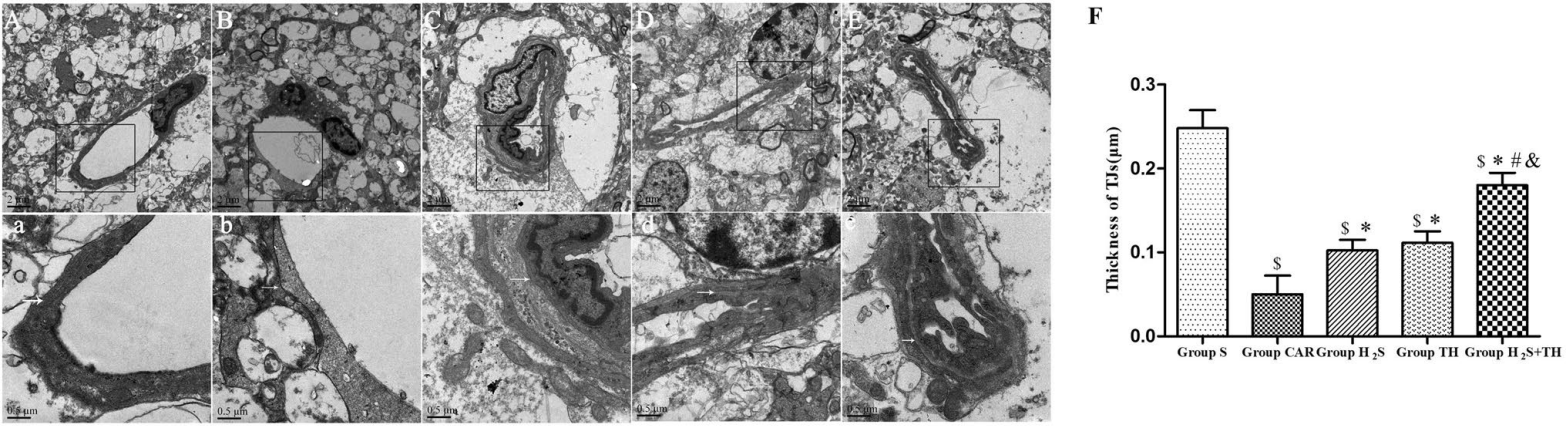
Ultrastructure alteration of BBB in the hippocampus of rats at 24 h after resuscitation or sham operation. The representative transmission electron micrographs of BBB are displayed, the micrographs in a-e are the high magnification of the area inside the boxes in (**A**–**E**), respectively. Arrows indicate the location of the TJs. Scale bar = 2 μm (**A**–**E**) or 0.5 μm (**a**–**e**). **A**Group S, **B** Group CAR, **C** Group H_2_S, **D** Group TH, **E** Group H_2_S + TH. **F** Quantification of the width of the TJs of BBB. (μm, mean ± SD). ^$^*P* < 0.05 versus Group S, ^*^P < 0.05 versus Group CAR, ^#^*P* < 0.05 versus Group H_2_S, ^&^*P* < 0.05 versus Group TH

Tight junctions are an important part of the BBB. Ultrastructure of TJs in hippocampus was observed by transmission electron microscope at 24 h after ROSC or sham operation. As shown in Fig. 6, electron microscopic overview of the rat cerebral capillaries showed in Fig. 6(A-E and a-e), and the width of the TJs of cerebral capillaries were measured as shown in Fig. 6(F). The difference of the width of the TJs in groups was statistically significant (*F*_*(4,20)*_ = *236.8; P* < *0.05*). Sham group showed an intact TJs, on the contrary, only a residual TJs was visualized in CA group. Compared to sham group, The average width was significantly decreased in the CAR group, H_2_S group, TH group and H_2_S + TH group (*P* < 0.05). Compared to CAR group, H_2_S group, TH group and H_2_S + TH group showed a relatively intact TJs and the average width was increased (*P* < 0.05), and width in the H_2_S + TH group was increased more than the H_2_S group (*P* < 0.05) or TH group (*P* < 0.05). These data demonstrated that ultrastructure of the BBB was incompletely injured in CA rats, and preserved by H_2_S or hypothermia treatment. Moreover, the combination of H_2_S with hypothermia showed a better protective effect.

## Discussion

The key findings of the study were that: CA resulted in brain edema and BBB disruption; H_2_S or hypothermia treatment diminished brain edema, decreased the permeability and preserved the structure of BBB during the early period of CA and resuscitation, improved the neurologic function, increased the 7-day survival rate after resuscitation; the combination of hypothermia and H_2_S treatment was more beneficial than that of hypothermia or H_2_S treatment alone.

Sudden CA is an important cause of death worldwide, many individuals who survive CA experience long-term disabilities [1, 27]. Ventricular fibrillation induced CA is a well characterized model to research the physiopathologic mechanism after CA and CPR, and the model is closer to clinical CA types. Previous research has found that both estrogen and progesterone could attenuate brain injury in rats, therefore, we used animals of only one sex here [28]. In our experiment, the physiological parameters demonstrated that we successfully produced a rat model of CA, and the overall successful recovery rate was about 85%. Hypothermia has been used to limit brain injury in certain clinical settings and animal models of brain insult, and recommended by international guidelines for CA survivors [27]. However, hypothermia by itself has not been consistently shown to produce long-term neurologic benefits across all studies [11]. Therefore, new neuroprotective approaches and combination therapy are warranted in the setting of global brain injury associated with CA. In a rat model of cerebral ischemia, our group demonstrated that combination of hypothermia and sodium hydrosulfide treatment for resuscitation was more beneficial for improving neuron survival than that of hypothermia or sodium hydrosulfide treatment alone [29]. In this experiment, we found that injection of sodium hydrosulfide or implemented with hypothermia both improved the survival rate and neurological function after CA and CPR.

The restoration of blood flow after ischemia can lead to secondary injuries. Disruption of the BBB after resuscitation has been shown one of the major factors leading to brain damage [3, 5]. A study of CA patients treated with hypothermia found the poor neurological outcome group showed severe BBB disruption in 24 h, 48 h, and 72 h after resuscitation [5]. An inevitable consequence of BBB disruption is the increase in the permeability of the BBB and the subsequently formation of brain edema [30]. Disruption of the BBB is not an “all-or-nothing” phenomenon, different sizes of exogenous tracers have been used to estimate the magnitude of the BBB opening [25]. In our study, BBB permeability was evaluated by measuring the EB and FITC–dextran content extravasated in the brain. EB dye which binds to albumin is an imaging marker for protein permeability, and the extravasation of FITC-dextran was used to test both solute and ion permeability. In our model, we found CA increased the permeability of BBB at 24 h after resuscitation, which was similar to other studies [4, 18, 31]. However, we should also note that some other studies have not found BBB disruption after CA. Mizushima et al. showed that the permeability of the BBB is not altered at 24 h after CA in a mice model, CA was induced by a complete interruption of cardiac output caused by compression of the major cardiac vessels in the study [32]. Similarly, Tress et al. showed that the BBB is not permeable to small and large molecular weight substances during the first 24 h after asphyxial CA, in their experiment, asphyxia of 9 min was induced in postnatal day 16–18 rats [33]. Differences in models and species may have played a role in these different results. In our experiment, both hypothermia and H_2_S treatment decreased the EB and FITC-dextran penetration significantly. The formation of brain edema induced by cerebral ischemia has been fully demonstrated. With the permeability of BBB increased, the proteins and solutes and ions permeated into extracellular spaces, leading to vasogenic edema, thereby increased intracranial pressure, exacerbated the ischemic state [3]. Similarly, severe brain edema was observed in the cortex and hippocampus at 24 h after resuscitation, and both hypothermia and H_2_S treatment lightened the water content. Here, we also demonstrated that ischemia impaired the ultrastructure of BBB by electron microscopy, and both hypothermia and H_2_S treatment restored the integrity of BBB. These results indicate the efficiency of hypothermia and H_2_S as a therapeutic strategy for brain edema and BBB disruption in a CA and CPR rat model.

The neuroprotective effects of hypothermia following cerebral ischemia including affect pathways leading to excitotoxicity, free radical production, inflammation and apoptosis, furthermore, hypothermia could protect BBB integrity by reducing the extracellular protease expression and activity, stabilizing the biological membrane [34]. As the third novel gasotransmitter, H_2_S can permeate cell membranes freely [15]. Studies have found that H_2_S presented potent protective effects against cerebral injury after CA, which mainly involves preservation of BBB, anti-apoptosis, anti-inflammatory and anti-oxidant mechanisms [18, 19, 23]. Marutani et al. reported in vitro and in vivo ischemic injury model experiments, the protective effects of H_2_S are mediated by thiosulfate that is transported across cell membrane by sodium sulfate cotransporter [17]. Therefore, H_2_S treatment may be a feasible neuroprotective strategy for patients survived after CA, with and without concurent treatment with hypothermia. Tight junctions (TJs) constitute the junction complex of the BBB, which is the important component for the maintenance of structural and functional integrity of BBB. TJs degradation is a crucial step in ischemic BBB breakdown [3, 35]. Protein occludin was the first integral transmembrane protein identified that localized to tight junction composition, the level of occludin decreased will lead to increased permeability of BBB [3, 36]. The expression of MMP-9 is a well-established destructive mediator of BBB disruption in cerebral ischemia, and MMP-9 has been shown to degrade TJs proteins that make up the BBB, leading to edema formation [6]. Models of brain ischemia have shown hypothermia protects the BBB and prevents edema formation, specifically, inhibits the expression of proteases and prevents the activation of MMP [34]. Li et al. reported in a swine model of CA and CPR, mild hypothermia attenuates early brain oedema and BBB disruption, and this improvement might be with suppression of MMP-9 expression [12]. Increasingly studies indicate that H_2_S plays an important role in the regulation of MMP-9. Geng et al. showed that inhalation of 80-ppm H_2_S immediately after CPR attenuated BBB permeability and brain edema, and the benefits could be associated with suppression of MMP-9 expression [18]. Similarly, our study showed that the expression of MMP-9 was upregulated after CA, the degradation of occludin was serious, importantly, both hypothermia and H_2_S treatment decreased the MMP-9 expression, reduced the degradation of occludin, more importantly, the combination of hypothermia and H_2_S showed a better protective effect. These results indicated that the protective effect on BBB integrity of hypothermia and H_2_S may rely on the inhibition of MMP-9 and preservation of the occludin of BBB. It is likely that the same and different mechanisms involved in hypothermia and H_2_S neuroprotection may be effective at different times after global cerebral ischemia and, therefore, add to the profound effect that has been observed.

The optimal timing for induction of hypothermia, the optimal target temperature and duration of hypothermia remain uncertain. In our current study, we selected the rectal temperature (34℃) as target temperature, and chose a 6 h hypothermic period induced within 15 min of initiating reperfusion. Slow rewarming is also considered important in avoiding harmful systemic responses. Here, we chose the rate as approximately 1℃/h over 4 h. In our study, hypothermia treatment attenuated early BBB disruption and brain edema and improved neurological outcome. We note that multiple randomised controlled trials or systematic review and meta-analysis showed that pre-hospital therapeutic hypothermia after out-of-hospital CA does not improve rates of survival with good neurological outcome or overall survival compared to no pre-hospital therapeutic hypothermia [37–39]; and research suggested that cooling to 36 °C results in benefits similar to cooling to 32–34 °C [14]. Further research is warranted. H_2_S salts, sodium hydrosulfide (NaHS) is often used as donors in research. Study is generally considered that therapeutic range of H_2_S is relatively narrow, a lower concentration of H_2_S exerts protective effect while higher levels of H_2_S exposure exerts damage effect. In our earlier study, we injected NaHS intraperitoneal, and using a methylene blue colorimetric assay, we found the concentration of H_2_S in the hippocampus tissue was 1.7-fold higher than that of ischemia–reperfusion control group [29]. Here, we selected a bolus injection of NaHS (0.5 mg/kg) at the beginning of CPR, followed by a continuous infusion of NaHS (1.5 mg·kg^−1^·h^−1^) for 6 h based on a previous study with minor modification [23]. Our results were consistent with which Kida et al. reported, mice received Na_2_S (0.55 mg/kg) before CPR improved the neurological function and 10-day survival rate [40]. However, Knapp et al. sought to evaluate the impact of Na_2_S on core body temperature and neurological outcome after CA in rats, they found that, after 6 min of global cerebral ischemia, a bolus of Na_2_S (0.5 mg/kg) 1 min before the beginning of CPR, followed by a continuous infusion of Na_2_S (1 mg·kg^−1^·h^−1^) for 6 h, sulfide therapy was associated with only a short term beneficial effect on neurological outcome, furthermore, sulfide had been shown to have no additive effect of the spontaneous hypothermic reaction after CA [41]. In their experiment, CA seems to trigger a spontaneous hypothermic response in rats, and we should exclude the confounding effect of spontaneous hypothermia in the experiment. In a mouse model of CA, Shin Nakayama et al. demonstrated that post-CPR treatment with NaHS exerted neuroprotection in mice while maintaining a normal cranial temperature, indicating that NaHS-related neuroprotection is independent of the known protective effect of spontaneous hypothermia [42]. In contrast, we investigated whether sodium hydrosulfide treatment amplifies the effects of deliberate hypothermia in regard to the neurological function and survival, and BBB disruption and brain edema induced by CA and resuscitation. Recently, Sun et al. utilized a unique platform for targeted controlled release of H_2_S, based on mesoporous iron oxide nanoparticle, which can be targeted to brain, offers a new method for cerebral protection from ischemic injury, and may bring considerable benefits for CA patients [43].

There were some limitations in this study. Firstly, the results were obtained in healthy rats, however, many of the patients suffering CA usually have underlying diseases. Secondly, the duration of CA in our study was relatively shorter, the periods of CA are generally longer in both experimental and clinical settings. Longer insult times may lead to different results and the protection conferred by therapeutic hypothermia and/or hydrogen sulfide may not be apparent in longer insults. Thirdly, we did not evaluate the histological assessment in our study. Thus, it is unknown if the benefits described herein correlate with neurohistopathologic outcome. Fourthly, we used rectal temperature to servo control temperature, tympanic or pericranial temperature might be a better approach to maintain constant brain temperature. Fifthly, due to the cooling technical reasons, the experimental procedure could not be blinded, but samples analysis and neurological function evaluation were performed by masked investigators. Sixthly, in this experiment, we used pure oxygen during resuscitation, but high concentration of oxygen inhalation may lead to a large number of reactive oxygen species, and may further damage the neurons. Adjusting the oxygen concentration according to the oxygen saturation may be more feasible.

## Conclusion

This study showed that combination of hypothermia and H_2_S after resuscitation was more beneficial for attenuated BBB disruption and brain edema, and improved neurologic function and 7-day survival rate than that of hypothermia or H_2_S treatment alone. The protective effects were associated with decreased the expression of MMP-9, and preserved of the tight junction protein occludin. The finding suggested that combined use of hypothermia and H_2_S treatment during resuscitation of CA patients could be a potential strategy to improve clinical outcomes and survival rate.

## Supplementary Information

Below is the link to the electronic supplementary material.

Supplementary file1 (DOC 109 KB)

## Data Availability

The original contributions presented in the study are included in the article/supplementary material, further inquiries can be directed to the corresponding author.
