# Supplementary material for: Therapeutic Hypothermia Combined with Hydrogen Sulfide Treatment Attenuated Early Blood–Brain Barrier Disruption and Brain Edema Induced by Cardiac Arrest and Resuscitation in Rat Model
Source: Neurochem Res. 2022 Nov 24;48(3):967–79. doi: 10.1007/s11064-022-03824-5 (PMC9922226; doi:10.1007/s11064-022-03824-5)
Supplement: Supplementary file 1 — Supplementary file1 (DOC 109 KB) [file 11064_2022_3824_MOESM1_ESM.doc]

**Supplement Table 1**

**Table 1. Therapies during CPR and ROSC rate**

|  | CAR  (n=15) | H_2_S  (n=15) | TH  (n=15) | H_2_S+TH  (n=15) |
| --- | --- | --- | --- | --- |
| Duration of CPR(s) | 115±22 | 108±18 | 117±16 | 109±20 |
| Number of defibrillations | 2.1±0.2 | 2.3±0.3 | 1.8±0.4 | 1.9±0.3 |
| Epinephrine i.v.(μg) | 8.5±1.0 | 8.3±0.8 | 7.9±0.7 | 8.2±1.0 |
| ROSC rate, n (%) | 13 (86.7) | 14 (93.3) | 13 (86.7) | 14 (93.3) |

Duration of CPR, number of defibrillations, dosage of epinephrine until ROSC and the rate of successful resuscitation in CAR, H_2_S, TH, H_2_S+TH groups. There was no difference between the groups. CPR, cardiopulmonary resuscitation; ROSC, return of spontaneous circulation. Values are expressed as means ± SD.

**Supplement Table 2**

**Table 2.** Physiological variables at baseline, 10, and 30 min After ROSC

|  | | CAR  (n=15) | H_2_S  (n=15) | TH  (n=15) | H_2_S+TH  (n=15) |
| --- | --- | --- | --- | --- | --- |
| **Baseline** | | | | | |
| pH | 7.39±0.05 | | 7.40±0.04 | 7.39±0.03 | 7.39±0.05 |
| PaO_2_ | 96.70±8.55 | | 97.30±9.21 | 98.50±7.86 | 96.90±10.28 |
| PaCO_2_ | 38.20±3.86 | | 37.80±4.25 | 38.40±5.12 | 37.90±3.90 |
| BE(mmol/L) | 0.50±0.35 | | 0.75±0.22 | 0.65±0.30 | 0.61±0.33 |
| Hematocrit(%) | 42.20±0.90 | | 41.90±0.80 | 42.70±0.60 | 41.80±0.90 |
| Glucose(mmol/L) | 5.18±1.13 | | 5.08±0.98 | 5.21±1.02 | 5.16±0.88 |
| Lactate(mg/dL) | 0.98±0.31 | | 0.89±0.43 | 0.96±0.55 | 1.02±0.34 |
| HR(bpm) | 400.00±32.40 | | 409±27.80 | 408±30.40 | 405±28.90 |
| MAP(mmHg) | 118.20±11.45 | | 120.40±14.50 | 115±17.80 | 111±20.40 |
| **10 min after ROSC** | | | | | |
| pH | 7.18±0.07 | | 7.20±0.04 | 7.19±0.05 | 7.18±0.03 |
| PaO_2_ | 357.80±35.20 | | 363±31.60 | 377±28.40 | 383±35.30 |
| PaCO_2_ | 36.70±5.80 | | 37.80±4.20 | 36.80±5.20 | 37.20±6.10 |
| BE(mmol/L) | -7.82±3.10 | | -7.05±2.98 | -7.10±3.80 | -7.20±2.10 |
| Hematocrit(%) | 42.80±0.50 | | 42.10±0.70 | 42.90±0.80 | 42.50±0.50 |
| Glucose(mmol/L) | 10.80±3.68 | | 11.28±4.01 | 10.30±3.50 | 9.98±4.10 |
| Lactate(mg/dL) | 9.20±2.10 | | 8.98±2.32 | 8.41±1.80 | 8.35±2.60 |
| HR(bpm) | 377.20±36.80 | | 375.60±30.10 | 352.30±24.40 | 350.10±28.30 |
| MAP(mmHg) | 110.10±9.20 | | 113.50±10.80 | 110.10±11.20 | 108±12.20 |
| **30 min after ROSC** | | | | | |
| pH | 7.31±0.08 | | 7.33±0.04 | 7.35±0.05 | 7.36±0.04 |
| PaO_2_ | 366.60±28.20 | | 368.20±20.50 | 370.20±21.30 | 369.20±20.90 |
| PaCO_2_ | 39.80±3.80 | | 40.20±5.10 | 38.90±4.20 | 39.30±5.30 |
| BE(mmol/L) | -4.10±2.10 | | -3.90±2.40 | -4.40±1.88 | -3.95±2.08 |
| Hematocrit(%) | 45.30±1.20 | | 46.10±0.90 | 45.70±0.80 | 46.20±0.50 |
| Glucose(mmol/L) | 7.48±3.20 | | 7.60±2.90 | 6.80±2.10 | 6.88±3.10 |
| Lactate(mg/dL) | 7.20±2.22 | | 7.01±1.89 | 5.04±2.02^*^ | 5.10±1.98^*^ |
| HR(bpm) | 388.20±20.40 | | 380.10±18.20 | 371.60±22.40 | 370.10±15.30 |
| MAP(mmHg) | 108.20±10.20 | | 109.20±9.33 | 105.10±5.80 | 101.50±9.80 |

Comparison of the results of arterial blood gas analyses, HR and MAP at baseline, 10 and 30 min after ROSC. Data was represented as mean ±SD, and compared by repeated measurement data analysis of variance followed by Bonferroni correction for post hoc intergroup comparisons.

PaO_2_ = arterial oxygen pressure; PaCO_2_ = arterial carbon dioxide pressure; BE = base excess; HR = heart rate; MAP = mean arterial blood pressure; ROSC, return of spontaneous circulation

^*^*P* < 0.05 *vs* the CAR group.

**Supplement Figure 1**


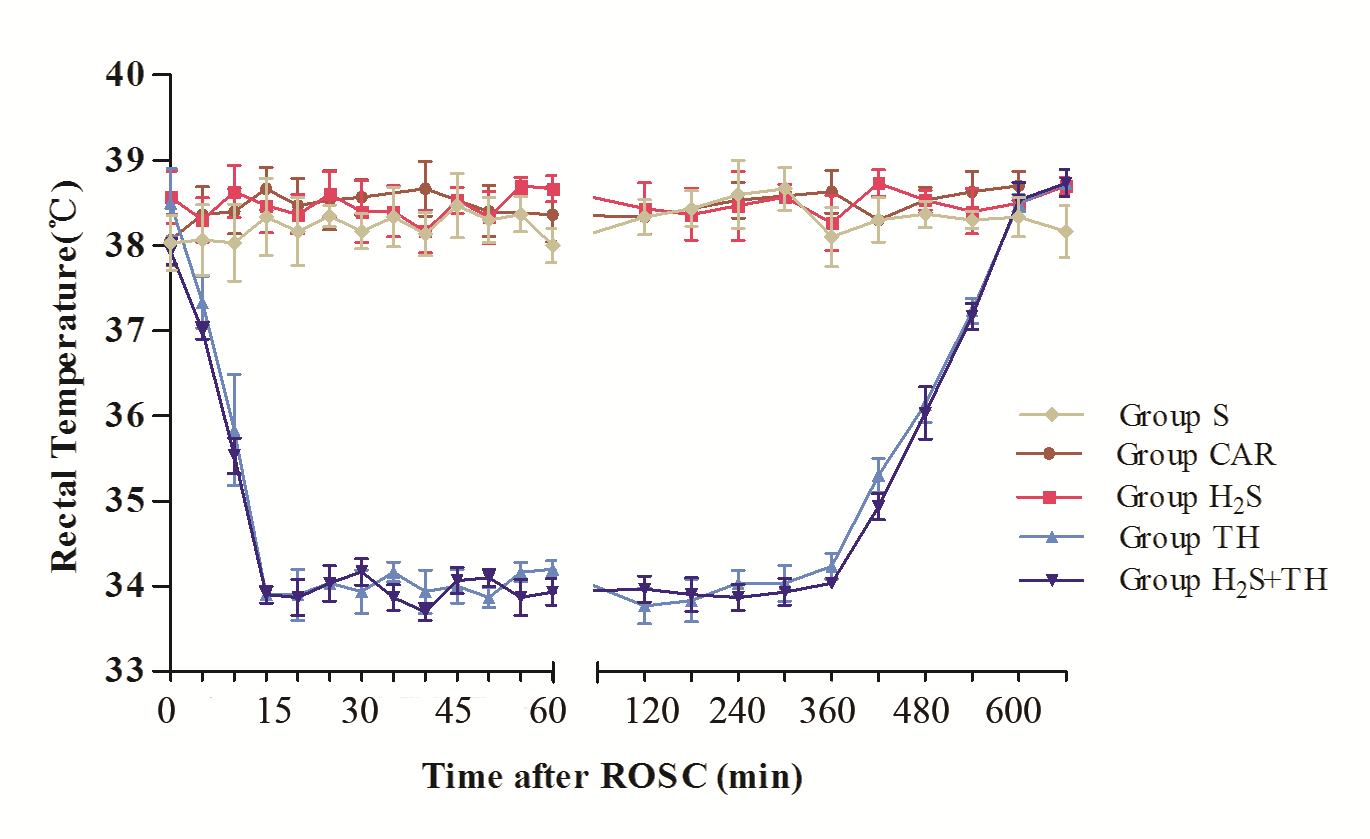


**Supplement Figure 1.** Time course of rectal temperature in rats. Rats were treated saline or NaHS at the beginning of resuscitation and were exposed to normothermia or whole-body hypothermia. During the period of re-warming, temperature was increased at approximately 1°C per hour. Values are shown as mean ± SD.
